# Fascination and Joy: Emotions Predict Urban Gardeners’ Pro-Pollinator Behaviour

**DOI:** 10.3390/insects12090785

**Published:** 2021-09-02

**Authors:** Ulrike Sturm, Tanja M. Straka, Alexandra Moormann, Monika Egerer

**Affiliations:** 1 Museum für Naturkunde—Leibniz Institute for Evolution and Biodiversity Science, Invalidenstraße 43, 10115 Berlin, Germany; Alexandra.Moormann@mfn.berlin; 2 Institute of Ecology, Technische Universität Berlin (TU), Rothenburgstraße 12, 12165 Berlin, Germany; Tanja.Straka@tu-berlin.de (T.M.S.); Monika.Egerer@tum.de (M.E.); 3 Department of Life Science Systems, TUM School of Life Sciences, Technische Universität München, Hans Carl-von-Carlowitz-Platz 2, 85354 Freising, Germany

**Keywords:** human-nature relationship, pollinator, nature conservation, socio-psychological factors, emotions, citizen science

## Abstract

**Simple Summary:**

The protection of pollinating insects depends on public support. Citizen science (CS) is frequently discussed as a way to promote interest and conservation action for pollinators. In cities, the drivers behind pollinator-friendly behaviour are largely unclear. We surveyed 111 community gardeners in Berlin and Munich, Germany, some of which were participating in a citizen science project. We created four different sets of generalized linear models to analyse how the gardeners’ pro-pollinator behaviour intentions and behaviour were explained by the gardener’s identity, emotions towards pollinators, attitudes towards pollinators and nature-relatedness. Respondents who were fascinated by pollinators, held positive attitudes, and felt joy about seeing pollinators reported intentions to protect or support pollinators. Similarly, joy about seeing pollinators was a predictor of participation in the CS project. We believe that CS may represent a pathway through which urban residents may become key actors in conservation projects within their nearby greenspaces.

**Abstract:**

The conservation of pollinators requires social understanding to catalyse restoration action. Citizen science (CS) is discussed as a way to promote interest and action for pollinating insects. Yet, the drivers behind pro-pollinator behaviour are largely unclear, especially in urban areas. To better understand public engagement in pollinator conservation, we studied urban community gardeners’ identity, nature-relatedness, emotions, and attitudes toward pollinators and their intentions to get involved in pro-pollinator behaviour in their gardening practice. We surveyed community gardeners in Berlin and Munich, Germany, some of which were participating in a citizen science project. In this scientific study, we created four different sets of generalized linear models to analyse how the gardeners’ pro-pollinator behaviour intentions and behaviour were explained by socio-psychological factors. The responses of 111 gardeners revealed that gardeners that were fascinated by pollinators, held positive attitudes and felt joy about seeing pollinators reported intentions to protect or support pollinators, suggesting that fascination and joy can be harnessed for research and conservation on pollinators. Similarly, joy about seeing pollinators predicted participation in the CS project. We believe that CS may represent a pathway through which urban residents may become key actors in conservation projects within their nearby greenspaces.

## 1. Introduction

Citizen science (CS), the involvement of citizens in scientific research and/or knowledge production, is being widely discussed in the scientific community and in policy as an instrument to address major global problems including insect population decline [1,2,3,4]. Insect pollinators have consequently become a widespread research topic for CS projects. CS engagement may include volunteers observing pollinators [5,6,7], collecting presence/absence data on nests [8], and projects investigating pollination [9,10]. Aside from a valuable source of data for researchers [11], CS is also proposed as an approach to increase interest in and appreciation of insect pollinators [12,13].

Changes in peoples’ (pro-)environmental behaviour (PEB) are also among the anticipated outcomes of CS participation [14,15]. PEB is gaining in importance, as people are increasingly important actors in pollinator conservation [16,17,18]. This is especially the case in the management of home or public gardens. Here, CS is promoted to support a change in the role of gardeners as stewards through pro-pollinator (or ‘pollinator-friendly’) habitat management [19,20]. In addition, participation in a pollinator CS project can be considered as PEB [21]. Despite this important link between CS participation and PEB, there are few insights about the factors that motivate gardeners’ pro-pollinator engagement.

Research on the motivations for participation and retention of participants in pollinator CS suggests that, while project dependent, educational motivations are the most important driver for volunteer participation [22,23]. Research on volunteering in an insect CS project showed the surveyed participants were driven by both altruistic (pro-social) and egoistic (self-serving) motivations, but rated the pro-social functions as more important for their engagement [24]. The methods also seem to be important, with complex projects having relatively low rates of retention and completion [9,23]. In a recent qualitative study on self-reported reasons for participating in pro-environmental CS activities, Tsybulsky [21] found that the influence of internal reasons (desire of social interaction, contribution, education, leisure as well as personal satisfaction and pleasure) were more important than external or demographic reasons to explain participation. While these assumptions are mostly based on self-reported motivations, we lack information on the underlying drivers of people’s behaviours.

Theoretical concepts such as Cognitive Hierarchy [25] propose that underlying drivers of people’s behaviours are attitudes and behavioural intentions. Following Cognitive Hierarchy, attitudes are defined as “an association, in memory, of an evaluation of an object” [26]. Behavioural intentions are close antecedents of behaviour [27] and often used as proxy of actual behaviour [28]. However, behavioural intentions do not necessarily predict actual behaviour [29], and other factors may influence PEB such as emotions [30], nature-relatedness [31] and identity [32].

Emotions influence mental processing including perceptions [33,34] and are often referred to as the root cause of human response to wildlife [35]. Furthermore, emotions related to behavioural intention are indicated to predict behaviour [36]. Environmental identity (to what extent one sees oneself as someone whose actions are environmentally friendly) plays an important role in PEB [37]. Van der Werff et al. [37] found that it is less likely that PEB is driven by intrinsic motivation (i.e., behaviour is satisfactory and enjoyable by itself [38]), but by a strong environmental identity, which makes people feel morally obliged to perform PEB. In addition, PEB is linked to human–nature connection [39], which can be described by peoples’ nature-relatedness, i.e., how people perceive their relationship with nature [31].

**Figure 1:**
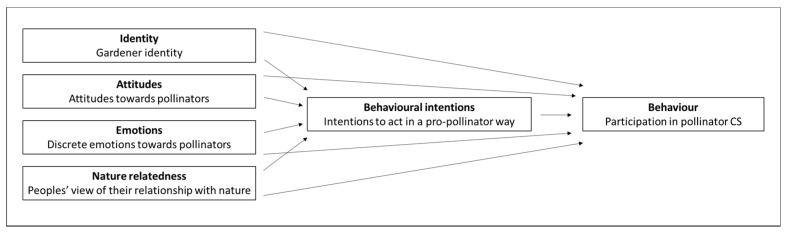
Adapted framework showing the relationship between the concepts identity, attitudes, emotions, nature relatedness, behavioural intentions and behaviour [25,30,37].

In relation to pollinators, Knapp and colleagues [18] showed peoples’ nature-connectedness, more specifically the diversity of interactions with nature, and their sense of being able to help pollinators are important predictors of people’s pro-pollinator actions. To a lesser extent, socio-psychological factors may be predictive of behaviour, including: attitude towards pollinators; knowledge of pollinators and conservation actions; perceived social pressure to help pollinators; and the environmentally friendly self-identity. To our knowledge, however, no previous work investigates multiple socio-psychological factors in relation to behaviour towards pollinators, including participation in pro-pollinator CS. Our work relates socio-psychological factors hypothesized to explain PEB-intentions and -behaviours towards pollinators (Figure 1), in our study, of urban gardeners.

We surveyed participants and non-participants of a wild pollinator CS project in community gardens in Berlin and Munich, Germany to investigate how identity, nature-relatedness, emotions and attitudes may predict participation in the CS project as a proxy for pro-pollinator behaviour and their intentions to get involved in pro-environmental behaviour. With this, we aim to better inform public engagement in pollinator conservation.

## 2. Materials and Methods

**Figure 2:**
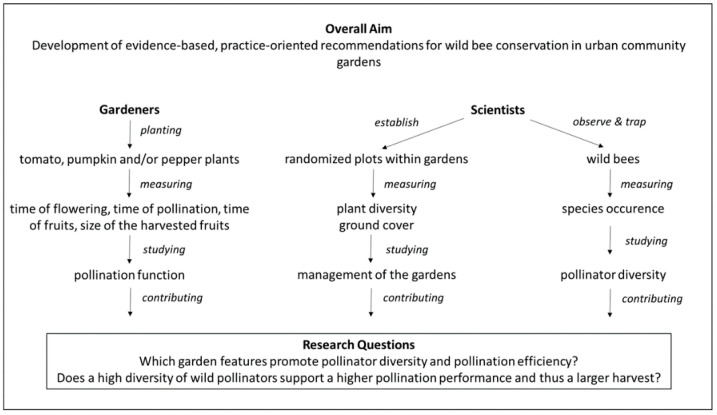
Overview of the ongoing pollinator CS project in community gardens in Berlin and Munich, Germany, detailing the collaboration of gardeners and scientists in urban ecology research for urban wild pollinator conservation.

We surveyed urban gardeners in 18 community gardens in Berlin, Germany and 15 community gardens in Munich, Germany. These gardens were part of an ongoing CS research project around wild pollinators and pollination function. In the project gardeners were asked to observe the pollination function over the course of the growing season of selected tomato, pumpkin and/or pepper plants by documenting when their plants (1) flowered, (2) were pollinated (closed flowers), and (3) had fruits. Gardeners measured the size of the fruit upon harvest. At the same time, researchers documented the management of the gardens (plant diversity, ground cover) and the wild pollinators in the gardens three times over the summer. Scientists and gardeners used different methods to study together the effect of garden features on pollinator diversity and pollination efficiency in the community gardens (Figure 2). The project initially started in Berlin in 2020 and was expanded to Munich in 2021 using the same CS methods to gather more comprehensive results. In 2020, 44 participants submitted data at the end of the gardening season. Exchange and contact between the gardeners and scientists took place mainly online due to the Corona pandemic in three organized Q&A sessions and during the field research on plant and pollinator diversity.

The survey questionnaire was distributed in online and in paper format after multiple project introduction and training meetings (April/May 2020 in Berlin, April/May 2021 in Munich) with the gardeners of the community gardens. In addition, non-participating gardeners in particular were surveyed in the course of the respective first project season until November 2020 in Berlin and until July 2021 in Munich. The survey instrument was delivered in German and took between 15 and 20 min to complete. Participation was anonymous, voluntary and questions could be skipped.

We measured the following variables in the survey instrument: nature-relatedness, emotions, attitudes, gardeners’ identity and behavioural intentions (Table 1). We gave the surveyed a short introduction with an illustrated overview showing that honeybees, wild bees, bumblebees, hoverflies, butterflies, wasps, flies and beetles are pollinators. To measure discrete emotions towards pollinators, we selected six single items of discrete emotions that relate to the concept of basic emotions [40] and are often studied in relation to wildlife: joy, fear, disgust, interest, fascination, anger and compassion [41,42]. To assess attitudes towards pollinators, we developed three items based on the definition of Fazio et al. [43] as in an ‘association, in memory, of an evaluation of an object’ attitudes to: (i) I like pollinating insects; (ii) I like pollinating insects in my garden; (iii) I think pollinating insects are interesting animals; and (iv) I think that pollinating insects are worth protecting. Specific identities are likely to be linked to behaviours related to identity [32,33]. Therefore, we focused on gardener identity based on their reported main reasons for urban gardening [44,45]: (i) gardening for food production; (ii) nature conservation/engagement, (iii) and social capital. Here we asked gardeners to report their level of agreement on a 5-point Likert scale to the following question “I consider myself as a gardener who appreciates …” with three items: (1) large harvest, (2) nature/species conservation, (3) social interactions. To assess PEB specifically concerning pollinator conservation, we assessed four items, which were adapted from Jacobs and Harms [46]. We measured the following behavioural intentions to get involved in pollinator conservation on a 5-point Likert scale: learning, implementing measures for pollinating insects, motivating others, and donating money. Following the same premise as Tsybulsky [21] we considered participation in the wild pollinator CS project as PEB.

Respondents indicated whether they were participating in the CS project before answering the questionnaire. Depending on participation or non-participation, we asked an open question about the motivation for participation, respectively the reasons for non-participation. For socio-demographics, respondents indicated, among other things, their age, gender and the size of the place they grew up.

The developed survey instrument was tested in a pilot with N = 12 people from different socio-demographic backgrounds and adapted when necessary (e.g., items were not clearly understood).

### Analysis

The analysis was performed in the R Statistical Environment (R version 4.0.2). We calculated the internal consistencies of latent constructs (i.e., attitudes, nature-relatedness and behavioural intentions) using Cronbach’s α. Emotions were single items and consequently, no internal consistency needed to be calculated. The latent constructs attitudes (Cronbach’s α = 0.78), nature-relatedness (Cronbach‘s α = 0.82), and behavioural intentions (Cronbach’s α = 0.84) showed acceptable internal reliability and consequently, all items were used for further analyses. For each latent construct, we calculated average scores of the associated items as composite indices. The composite index of attitudes were the averaged four items measuring attitudes towards pollinators, of nature-relatedness the averaged six items of the NR-6 and of behavioural intentions the respective averaged four items about intentions to get involved in pollinator conservation. We used participation in the CS as a proxy for behaviour. Thus, we included a binary response variable indicating participation (1) and non-participation (0) (hereafter as behaviour).

We created four different sets of generalized linear models to analyse how the (latent construct of) gardeners’ pro-pollinator behaviour intentions and behaviour were explained by the gardener’s identity, emotions towards pollinators, attitudes towards pollinators and nature-relatedness. We separated cognitive concepts (e.g., attitudes) and emotions in our models given that the Cognitive Hierarchy focuses on cognitive processes and does not include emotions. Consequently, and given that the sample size did not allow us to include all variables in one model, we ran each model separately. First, with behaviour as binary response variable (binomial distribution) and behavioural intentions, identity, attitudes and nature-relatedness as explanatory variables. Second, again with behaviour as response variable and the six emotions as explanatory variables. Third and fourth, we used behavioural intentions (quasipoisson distribution to take into account the underdispersed count data) as response and again, identity, attitudes and nature-relatedness as well as the six emotions as explanatory variables, respectively.

Additionally, we analysed the motivation for participation and the reasons for not participating using qualitative content analysis [48]. The coding categories were based on recent literature on participation in pollinator CS [21,22,23,24]. Two researchers discussed and adopted the coding categories to achieve a mutual understanding and sufficient coding consistency.

## 3. Results

A total of 111 gardeners answered the survey questionnaire—approximately one fifth of the gardeners of the 32 community gardens. More than half (59%) of the respondents identified as female, 37% as male, and 4% of the gardeners did not specify. The age of the respondents ranged from 21 to 76 years (mean age 42.4 years, SD = 15.1). About a third of the total sample (N = 41) participated in the wild pollinator CS project. In comparison, more of the CS participants identified as female and this group of respondents was on average slightly older (see Table 2).

### 3.1. Identity, Emotions, and Attitudes in Relation to Gardener Behavioural Intentions and Behaviour

Attitudes towards pollinators and the emotions of “fascination” and “joy” (when seeing a pollinator on a plant) were found to have positive predictive potential for behavioural intentions to get involved in pollinator protection, whereas “anger” had a significant negative effect on behavioural intentions (Table 3). Furthermore, “joy” when seeing a pollinator on a plant was found to have positive predictive potential to join the CS project.

### 3.2. Self-Reported Reasons for Participation and Non-Participation in Pollinator CS

The most frequently self-reported motivation for participation was education with a focus on knowing more about pollinators, interest in the overall project and contribution to something meaningful as nature conservation (Table 4). Leisure, personal satisfaction, and pleasure as well as social interactions were less frequent reasons.

The non-participating gardeners described unawareness of the project and lack of time as the most common reasons for not participating (Table 5). One person responded that the protocol’s difficulty prevented them from participating; another person stated their fear of insects as the reason.

## 4. Discussion

Our study shows the importance of emotions for pro-pollinator behaviour, providing new insight into the role of emotions in public engagement in nature conservation. In line with other research in gardens [18] and the Cognitive Hierarchy of human behaviour [25], we confirm that both attitudes and emotions towards pollinators have predictive potential on behavioural intentions on pro-pollinator behaviour. Perhaps more importantly, we found that emotions (i.e., joy about seeing pollinators) had predictive potential on behaviour—here, the participation in a wild pollinator CS project. Thus, our results argue for an empathetic approach for pollinator conservation initiatives to engage and empower the public for conservation action [18,49].

### 4.1. Positive Emotions Predict Pro-Pollinator Behavioural Intentions and Action

The surveyed gardeners who are fascinated in pollinators and who feel joy towards pollinators reported intentions to protect or support pollinators. In addition, gardeners who feel joy towards pollinators demonstrated action by participating in the wild pollinator CS project. This confirms the importance of understanding positive emotions in relation to wildlife to foster co-existence and to avoid a negativity bias [50]. Fear and disgust are among the common emotions in insect-focused research [51]. Urbanization tends to increase disgust towards insects since they are mostly experienced indoors and perceived as pests or a nuisance [52], and urban residents tend to have less knowledge about insects due to disconnection to nature [53]. We also found anger towards pollinators to negatively affect intentions to engage in pro-pollinator behaviour among surveyed gardeners. However, our results suggest moving beyond these negative emotions to encourage pro-pollinator behaviour.

We believe that integrating emotions such as fascination and joy create new opportunities for heightening public engagement for pollinator conservation. Furthermore, future CS and conservation research investigations should closely assess the mechanisms behind the role of fascination and joy. Experiencing joy both positively affected intentions and behaviour, showing that select emotions can consistently predict both up- and downstream factors in Cognitive Hierarchy. However, fascination did not lead to action in the form of participation in the wild pollinator CS project. Knapp et al. [18] found that different behaviour is driven by different factors, so fascination could be predictive for other pro-pollinator actions beyond participating in a wild pollinator CS project. Therefore, we propose that future research should study emotions in regard to different pro-pollinator activities. Mitigating emotions such as fear, anger, and disgust should also be integrated into pro-pollinator activities such as CS projects to reduce barriers to participation.

### 4.2. Motivations to Participate in Wild Pollinator CS Are Diverse

Our results are in line with other research showing that gardeners are motivated to participate in pollinator CS for numerous reasons, both altruistic and egoistic [21,24]. Overall, the self-reported reasons and the socio-psychological factors that predicted the participation in the wild pollinator CS project pointed in a similar direction: enthusiasm for pollinators is the most important reason for engagement. Interest and the desire for education were among the most frequent self-reported motivations for participation, in line with previous studies [22]. Similar to Moczek, Nuss, and Köhler [24], the altruistic reason to contribute to something meaningful such as nature conservation was also an important driver to participate in our wild pollinator CS project. This shows CS to be an opportunity to do something for pollinators. However, the self-reported motivations were rather general in comparison. We think that mixed-methods design, in particular the use of qualitative interviews, will yield further valuable insights into the motivations of pro-pollinator behaviour and participation in CS.

In addition, the self-reported reasons show that motivation for CS participation goes beyond conservation and content interest. Collaborating with and supporting scientific research were also important motivations for more than a third of the respondents. This finding is in line with arguments that CS has great potential for promoting public understanding of science [15,54,55]. However, whether and how this potential can be realised remains to be determined. The self-reported barriers to participating in wild pollinator CS were almost exclusively at a practical level, contributing to our understanding of challenges in pollinator conservation [18]. We think that this issue of motivation not to participate should be given more attention in order to engage more people and more diverse participants in conservation [55]. There is a need for more detailed analysis on which drivers prevent action as well as various sound and easy to implement measures for pro-pollinator actions as differences in participation could be due to the nature and tasks of projects [23].

We therefore suggest that understanding participants and non-participants, as well as the influence of the task, scope, and context of pro-pollinator activities such as CS projects, should be more thoroughly included in research on participation and project design. Chase and Levine [56] argue that the potential of CS to enhance conservation can only be achieved through a deeper insight into who participates and the ability of these programmes to have a real impact on attitudes and behaviour. We propose a stronger focus on socio-psychological factors that influence behaviour.

### 4.3. Limitations

Our study has a number of limiting factors. The sample size is relatively small with the risk of self-selection bias. In addition, participants and non-participants were surveyed at different times during the course of the project, but always in the first year of implementation. This may have created a bias towards a greater awareness and value of pollinators. Furthermore, we do not have any information on the quality and quantity of respondents’ participation in the CS project e.g., how many respondents submitted complete data. We can therefore not make any statements as to which factors may influence long-term behaviour (e.g., persistence, and intensity), and project retention.

## 5. Conclusions

Our findings show the importance of positive emotions such as fascination and joy for pollinator conservation projects and PEB, as both predicted behavioural intentions and pro-pollinator behaviour such as the involvement in a wild pollinator CS project. New adapted formats tailored for urban gardeners are still needed to engage, inspire and empower the public for pollinator conservation in cities. We suggest that citizen science may represent a pathway through which urban gardeners may not just learn more about pollinators but can be key actors in conservation projects within their nearby greenspaces.

## Figures and Tables

**Table 1:** Overview of studied variables and used measurements in the survey instrument.

Variable	Measurement	References
nature-relatedness	six-question short-form version of the nature-relatedness scale (NR-6)	[47]
discrete emotions towards pollinators: joy, fear, disgust, interest, fascination, anger	Rating of feelings, when seeing a pollinator on one of their garden plants on a 7-point scale	[40,41,42]
attitudes towards pollinators	Rating of agreement on a 5-point Likert scale to the following statements: I like pollinating insects, I like pollinating insects in my garden, I think pollinating insects are interesting animals, I think that pollinating insects are worth protecting	[43]
gardener identity	Rating of agreement on a 5-point Likert scale to three motivations for urban gardening	[37,44,45]
behavioural intentions	Rating of agreement on a 5-point Likert scale to get involved in pollinator conservation by learning, implementing measures for pollinating insects, motivating others, and donating money	[46]
PEB	participation in CS pollinator project	[21]

**Table 2:** Demographics of respondents (N = 111) and respondents, who participated in the pollinator CS project (N = 41).

	Total Respondents (N = 111)	Respondents, Who Participated in CS Project (N = 41)
gender	59% female,	67% female
	37% male	31% male
	4% no information	2% no information
age	21 to 76 years	24 to 74 years
	mean age 42.9 years, SD = 15.1	mean age 45.4 years, SD = 15.4
size of city they grew up	21% < 2000 inhabitants	32% < 2000 inhabitants
	21% 2000 to 50,000 inhabitants	27% 2000 to 50,000 inhabitants
	14% 50,000 to 200,000 inhabitants	7% 50,000 to 200,000 inhabitants
	36% > 200,000 inhabitants	32% > 200,000 inhabitants
	7% no information	2% no information

**Table 3:** Predictive potential of the constructs (identity, emotions, attitudes and nature-relatedness) to get involved in pollinator protection and predictive potential of these constructs in the participation of a CS project based on generalized linear models (effect sizes/-SE, GLMs), Model 1 and 2 included behavioural intention and model 3 and 4 participation as response variable in the models. Significance levels shown by * *p* < 0.05, ** *p* < 0.001, *** *p* < 0.0001.

	Behavioural Intention to Get Involved in Pollinator Protection Est. ± SE (*p*-Value)	Participation in Pollinator CS Project (Behaviour) Est. ± SE (*p*-Value)
Model 1 and 3		
behavioural intention	-	0.57 ± 0.32 (0.08)
identity food production	0.03 ± 0.02 (0.19)	0.01 ± 0.22 (0.98)
identity nature conservation	0.02 ± 0.04 (0.74)	0.91 ± 0.49 (0.06)
identity social capital	0.03 ± 0.03 (0.23)	−0.33 ± 0.27 (0.22)
attitude	0.17 ± 0.05 *** (<0.001)	0.07 ± 0.53 (0.89)
nature-relatedness	0.08 ± 0.04 (0.06)	−0.41 ± 0.42 (0.33)
Model 2 and 4		
joy	0.08 ± 0.03 * (0.02)	0.95 ± 0.35 ** (0.007)
fear	0.04 ± 0.03 (0.19)	−0.11 ± 0.37 (0.77)
disgust	0.001 ± 0.05 (0.97)	0.002 ± 0.49 (1.00)
interest	−0.005 ± 0.03 (0.86)	−0.02 ± 0.29 (0.94)
fascination	0.08 ± 0.03 * (0.02)	−0.19 ± 0.30 (0.54)
anger	−0.15 ± 0.06 * (0.02)	0.16 ± 0.59 (0.79)

**Table 4:** Self-reported reasons to participate in the pollinator CS project, multiple answers possible, N = 39.

Self-Reported Reasons to Participate	Respondents in %
interest in the overall project, also in relation to its relevance for the garden	46
interest in and learning about pollinators/wild bees	54
interest in and contributing to scientific research	38
becoming active and participating in something meaningful as nature conservation	44
leisure, fun, social interaction	10

**Table 5:** Self-reported reasons not to participate in the pollinator CS project, multiple answers possible, N = 25.

Self-Reported Reasons Not to Participate	Respondents in %
unawareness of the project	52
lack of time	44
difficult protocol	4
fear of insects	4

## Data Availability

The data presented in this study are available on request from the corresponding author. The data are not publicly available as the participants provided the data specifically for the purpose of research in the context of the pollinator CS project.
